# Physical activity in obesity and metabolic syndrome

**DOI:** 10.1111/j.1749-6632.2012.06785.x

**Published:** 2012-11-21

**Authors:** Barbara Strasser

**Affiliations:** Department of Medical Sciences and Health Systems Management, Institute for Nutritional Sciences and Physiology, University for Health Sciences, Medical Informatics and TechnologyHall in Tirol, Austria

**Keywords:** behavior, dose response, metabolic risk, obesity, physical activity, resistance training

## Abstract

Biological aging is typically associated with a progressive increase in body fat mass and a loss of lean body mass. Owing to the metabolic consequences of reduced muscle mass, it is understood that normal aging and/or decreased physical activity may lead to a higher prevalence of metabolic disorders. Lifestyle modification, specifically changes in diet, physical activity, and exercise, is considered the cornerstone of obesity management. However, for most overweight people it is difficult to lose weight permanently through diet or exercise. Thus, prevention of weight gain is thought to be more effective than weight loss in reducing obesity rates. A key question is whether physical activity can extenuate age-related weight gain and promote metabolic health in adults. Current guidelines suggest that adults should accumulate about 60 minutes of moderate-intensity physical activity daily to prevent unhealthy weight gain. Because evidence suggests that resistance training may promote a negative energy balance and may change body fat distribution, it is possible that an increase in muscle mass after resistance training may be a key mediator leading to better metabolic control.

## Introduction

Both overweight and obesity are characterized by the accumulation of excessive levels of body fat. Intraabdominal (viscera fat) increases by over 300% between the ages of 25 and 65 years,^1^ and this creates an increased risk for the development of heart disease, hypertension, type 2 diabetes (T2D), and some cancers.^2^,^3^ The underlying reasons for the increased risk are not well understood, although it is likely that an age-related decrease in physical activity (PA) contributes to this problem. Lifestyle modification—specifically changes in diet, PA, and exercise—is considered to be the cornerstone of obesity management.^4^ However, it is important to distinguish between active lifestyle and physical fitness. While PA refers to any movement produced by skeletal muscles that expends energy, exercise improves the efficiency and capacity of the cardiorespiratory system and muscular strength associated with health and functional capacity.^5^ Overall, PA is associated with many health-related benefits, including a reduced risk of developing several chronic diseases such as obesity,^6^ cardiovascular disease (CVD),^7^ T2D,^8^ metabolic syndrome (MS),^9^ and cancer.^10^ PA guidelines for healthy individuals have evolved over the last decade for the purpose of preventing the onset of disease (i.e., primary prevention).^11^ Recommendations for PA and health have included 30 min/day (or more) of at least moderate-intensity PA on most days of the week with respect to cardiovascular benefits.^12^,^13^ However, with the increasing prevalence of overweight and obesity (66.3% of adults in the United States are currently overweight (BMI ≥ 25 kg/m^2^) and 35.5% are obese (BMI ≥ 30 kg/m^2^)),^14^ it is important to provide guidance to obese individuals on how much PA is needed to promote metabolic health and to lose weight. Major health organizations, such as the International Association for the Study of Obesity (IASO)^15^ and the American College of Sports Medicine (ACSM), consistently support the need for more than 150–250 min/week of moderate-intensity PA to prevent weight gain.^16^ However, there is currently a lack of guidance for obese individuals on feasible strategies for weight loss and prevention of weight regain. The purpose of this review is (1) to explore the relationship of sedentary behavior with major health outcomes, and (2) to illustrate the potential role of PA and supervised exercise interventions (aerobic and resistance training (RT)) in the prevention and treatment of obesity and MS risk factors. Considering the benefits of exercise training on changes in metabolic risk factors among obese individuals, we ask an important question: How much exercise is needed to confer such benefits?

## Sedentary behavior: a new health risk

**Figure 1:**
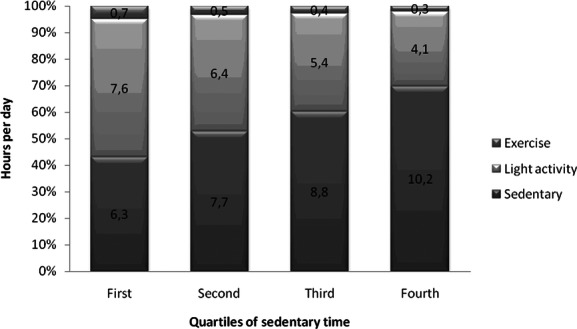
Distribution of time (hours/day) spent in sedentary, light-intensity physical activity and exercise according to quartiles of sedentary time from the U.S. National Health and Nutrition Examination Survey (NHANES).^20^ Based on one week of accelerometer data in 1,712 adults, the stacked column graphs show the allocation of walking hours spent sedentary, in light activity, and in exercise, from the lowest (first) to the upper (fourth) quartile of overall sedentary time. Adapted and modified from work by Owen *et al*.^21^

Sedentary behavior (too much sitting) is associated with deleterious health outcomes, which differ from those that can be attributed to a lack of moderate–vigorous PA (MVPA; too little exercise).^17^,^18^ This has led to the field of *sedentary physiology*, which may be considered as separate and distinct from exercise physiology. In this paper, PA refers to activities of at least moderate intensity (3–6 metabolic equivalent tasks (METs); light activity includes all movements <3 METs and >1.5 METs; and sedentary behaviors are considered those requiring ≤1.5 METs).^19^ Based on one week of accelerometer data from the U.S. National Health and Nutrition Examination Survey (NHANES),^20^ the vast majority of daily nonsleeping time is spent in either sedentary behavior (58%) or light-intensity activity (39%), and only 3% in exercise time (Fig. 1).^21^ Most of the variance in sedentary time is due to the change in the proportion of time spent in light-intensity activity. For example, sedentary time increases from 6.3 h in quartile 1–10.2 h in quartile 4, a 62% increase with nearly all of the sedentary time coming out of the block of light activity. Importantly, individuals can achieve high levels of MVPA and still exhibit high levels of sedentary behavior. This phenomenon, dubbed *the active couch potato*, distinguishes sedentary behavior as a unique health risk since it has been associated with impaired cardiometabolic health.^22^,^23^ Accordingly, it was the aim of a very recent study to describe the independent and combined effects of MVPA and sedentary behaviors (television viewing, overall sitting) on cause-specific mortality.^24^ In the National Institutes of Health (NIH)-AARP Diet and Health Study, 240,819 U.S. adults (aged 50–71) were examined who did not report any cancer, CVD, or respiratory disease at baseline. Time spent in sedentary behaviors was positively associated with mortality, and participation in high levels of MVPA did not fully mitigate health risks associated with prolonged time watching television. Even among adults reporting high levels of MVPA (>7 hours/week), high amounts of television viewing (≥7 hours/day) remained associated with increased risk of all-cause (HR: 1.47; 95% CI: 1.20, 1.79) and cardiovascular mortality (HR: 2.00; 95% CI: 1.33, 3.00) compared with those reporting the least television viewing (less than one hour/day).^24^ These findings indicate that both sedentary behaviors and MVPA are associated with mortality, However, it seems that sedentary behavior, as distinct from a lack of MVPA, has independent and qualitative different effects on human metabolism, physical function and health outcomes and thus should be treated as a unique construct.^19^,^23^,^25^

## Inactivity physiology

**Figure 2:**
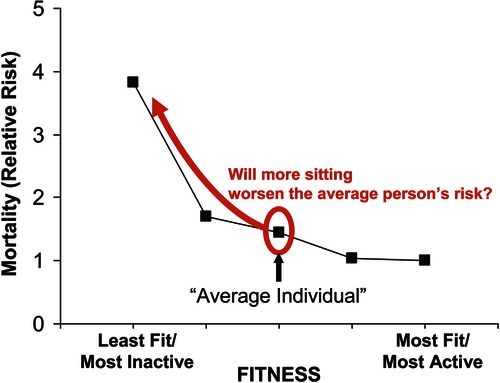
Fitness–mortality curve. Relationship between cardiorespiratory fitness and mortality in 13,344 middle-aged men and women. The question is whether the typical person who already does not perform structured exercise regularly will have increased risks of metabolic diseases in the coming years as a result of too much sitting. Too little exercise and too much sitting could push the fitness–mortality curve upward or shift it to the left, where there is the most risk for disease. Source from work by Hamilton *et al*.^25^

A major question raised by the inactivity physiology paradigm is whether the typical person who already does not perform structured exercise regularly will have increased risks of metabolic diseases in the coming years as a result of too much sitting. As described by Hamilton *et al*.,^25^ too little exercise and too much sitting could push the fitness–mortality curve upward or shift it to the left, where there is the most risk for disease (Fig. 2).^26^–^28^

Recent studies have documented deleterious associations of reported television viewing time and overall sedentary time with central adiposity, fasting triglyceride levels, and markers of insulin resistance that are independent of both central adiposity and exercise time.^23^,^29^–^31^ With prolonged periods of sitting, fewer skeletal muscle contractions may result in reduced lipoprotein lipase (LPL) activity and clearance of triglycerides, reduced clearance of an oral glucose load, and less glucose-stimulated insulin secretion.^32^,^33^ Studies examining LPL regulation have shown that LPL activity decreases 10-fold locally in the oxidative muscle regions within hours after becoming inactive.^25^ Low-intensity PA produces a potent biochemical signal for activation of LPL activity and subsequently LPL-dependent plasma triglyceride uptake.^25^,^34^ In addition to LPL activity, several reports suggest that sedentary behavior affects carbohydrate metabolism through changes in muscle glucose transporter (GLUT) protein content. Studies have shown that denervation of skeletal muscle results in rapid decreases in both muscle GLUT-4 content and insulin-stimulated glucose uptake.^35^ Further, physical inactivity rapidly induces insulin resistance and microvascular dysfunction in healthy adults.^33^ Hamburg *et al*. examined the effect of five days of complete bed rest on metabolic health in 22 adult volunteers and reported a 67% greater insulin response to a glucose load (*P* < 0.001) following the five-day intervention.^33^ However, even minor increases in contractile activity can dramatically increase LPL activity, muscle GLUT content, and glucose tolerance in sedentary individuals.^25^,^36^ Low-intensity PA does not affect all skeletal muscles to the same degree, but instead affects the recruitment and metabolic responses locally in slow oxidative muscle fibers that are responsible for the rapid impairment of plasma lipoprotein and glucose metabolism after PA.^32^ Importantly, reducing sedentary time has a much greater effect on LPL regulation than adding vigorous exercise training on top of the normal level of nonexercise activity.^25^ Thus, the average nonexercising person may become even more metabolically unfit in the coming years if they sit too much.

## Epidemiologic evidence

Increasing evidence from prospective studies indicates detrimental associations between excessive sitting, and television viewing time, in particular, and cardiovascular risk factors, which are independent of PA and other relevant covariates. These include weight gain and incident obesity,^37^,^38^ dyslipidemia,^39^ hypertension,^40^ and insulin resistance and T2D.^22^,^37^,^41^ A dose–response relationship was recently observed between time spent in sedentary behaviors (e.g., TV viewing time, sitting in a car, overall sitting time) and all cause and CVD mortality.^17^,^18^,^42^ The study by Hu *et al.* using data from the Nurses’ Health Study provides key evidence regarding the relationship between sitting and health outcomes, including obesity.^37^ A total of 50,277 women, who were not obese at baseline, were followed over a six-year period. In analyses adjusting for other lifestyle factors, including diet and PA, each two hours/day increase in TV viewing time was associated with a 23% (95% CI: 17–30%) increase in obesity and a 14% (95% CI: 5–23%) increase in T2D.^37^ A similar finding was observed in 37,918 participants of the Health Professional's Follow-up Study, where, independent of PA, each two hours/day increase in TV-viewing time was associated with a 20% increase in the risk for T2D.^22^ TV-viewing time has also been associated with an increased risk of biomarkers of cardiometabolic risk,^29^,^43^ as well as the MS.^44^,^45^ The EPIC Norfolk Study, a population-based cohort of 12,608 men and women (aged 61.4 ± 9.0), indicates that sedentary behavior (TV-viewing time) is associated with CVD, independently of PA energy expenditure and other confounding variables.^46^ Every one hour/day increase in TV viewing was associated with a 6% (95% CI: 3–8%) higher risk for total and nonfatal CVD and an 8% (95% CI: 3–13%) higher risk for coronary heart disease (CHD).^46^

In recent studies that have used accelerometer-derived measures to objectively assess sedentary and physically active time, high levels of adults’ sedentary time have been detrimentally associated with waist circumference, triglycerides, two-hour plasma glucose,^23^,^36^ and insulin.^47^ In a 6.5-year follow-up of the AusDiab study, each one-hour increment in TV time was found to be independently associated with an 11% (95% CI: 3–20%) and an 18% (95% CI: 3–35%) increased risk of all-cause and CVD mortality, respectively.^17^ Importantly, more breaks in sedentary time were beneficially associated with several of the outcomes.^48^ Thus, there may be metabolic health benefits of regular interruptions to prolonged sitting time, which may be in addition to the likely benefits of reducing overall sedentary time.

## PA, fitness, and obesity

Observational or cross-sectional data on the relationship among PA, cardiorespiratory fitness level, and body weight and obesity have shown an inverse association between these measures.^49^,^50^ Physically active and fit individuals are considerably less likely to be obese than physically inactive and unfit individuals. However, for most overweight people, it is difficult to lose weight permanently through diet or exercise. Thus, prevention of weight gain is thought to be more effective than weight loss in reducing obesity rates. A key question is whether increasing PA can mitigate age-related weight gain in adults. Even though the results are not entirely consistent, most population-based longitudinal studies have found that increasing PA attenuates gain in weight or waist circumference during midlife.^4^,^6^,^51^–^53^ Accordingly, an important question is, How much PA is required to prevent age-related weight gain?

## Prevention of weight gain

**Figure 3:**
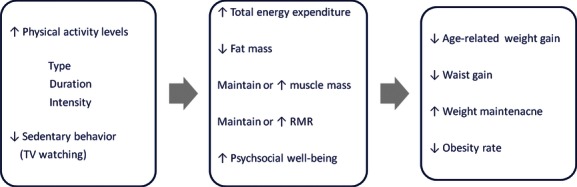
Potential pathways through which physical activity and sedentary behavior influence obesity rate and weight gain. Adapted and modified from work by Hu.^54^

Cumulative evidence from prospective cohort studies and randomized clinical trials indicates that PA and active lifestyle play an important role in weight management. The biological mechanisms by which PA prevents weight gain are multiple (including increasing total energy expenditure, reducing fat mass, maintaining lean body mass, and basal metabolic rate) and may depend on the type and intensity of PA (Fig. 3).^54^

The optimal amount of exercise needed to prevent weight gain in adults appears to vary by age, sex, and energy intake.^6^ Among male participants 40–75 years of age in the Health Professionals Follow-up Study, increasing vigorous exercise to 1.5 hours/week attenuated weight gain but was insufficient to offset it completely.^55^ In the CARDIA study—a cohort of black and white men and women, aged 18–30 years at baseline—the predicted weight change over a 10-year period associated with change in PA was four to five times larger in participants who were overweight compared with those who were not overweight at baseline.^53^ Further, increasing high-intensity exercise (requiring six MET hours) by two hours/week above baseline PA was needed to offset observed weight gain. Overall, the young adults needed to exercise an average of four to five hours/week to completely prevent weight gain.^53^ These estimates are in broad agreement with those from the Aerobics Center Longitudinal Study, which determined the relation between the average daily PA level (PAL) and five-year weight change in men at risk for weight gain.^56^ Increasing or maintaining a daily PAL at least 60% above the resting metabolic rate is necessary to maintain body weight in the middle-aged, which can be achieved by incorporating 45–60 min/day of exercise. Thus, the amount of PA required preventing weight gain may be higher than that recommended in current PA guidelines for prevention of chronic diseases (30 minutes or more of moderate-level activity on most days of the week).^12^,^13^ In 2009, the American College of Sports Medicine published a Position Stand that recommended 150–250 min/week of MVPA with an energy equivalent of 1,200–2,000 kcal/week to prevent a weight gain greater than 3%.^16^ However, there is increasing evidence that reducing sedentary behaviors such as prolonged TV watching is beneficial for weight control independent of the amount of exercise.^57^ Therefore, public health recommendations for prevention of obesity should encourage both, increasing PA but also reducing sedentary behaviors.

## Treatment for weight loss

There is sufficient evidence to conclude that PA interventions between 150 and 250 min/week, in the absence of dietary intervention, produce only modest weight loss (2–3 kg).^16^ The STRRIDE study underscored the minimal benefit of exercise alone for weight loss.^58^ Individuals who jogged the equivalent of 20 miles a week lost only 3.5 kg (SD: 2.8) at the end of eight months of training. Individuals who walked 12 miles a week at a moderate intensity lost only 1.1 kg (SD: 2.0).^58^ Thus, exercise training without dietary intervention has a relatively small effect on weight control. The lack of efficacy for exercise to promote weight loss may be in part due to the relatively low amount that has been used in exercise studies. Ross *et al*. showed that the energy expenditure of exercise has to be between 500 and 700 kcal/day to provide a weight loss of approximately 6 kg in women and 8 kg in men within 12 weeks.^59^,^60^ Fogelholm *et al*. reviewed the effects of walking (150–225 min/week) on weight loss in obese participants with or without low-energy diet (1,200–1,600 kcal/day).^61^ The mean weight reduction during three to six months when walking was added to diet was numerically (by 0.3–2.1 kg) but not significantly better than the diet-only group.^61^ Unfortunately, most of the exercise studies for weight reduction did not measure the total caloric deficit and its correlation with the loss of body mass. When the energy deficit imposed by diet-only and diet plus PA interventions are similar, weight loss, and/or percentage change in body weight are similar.^62^

A number of trials have demonstrated that the combination of exercise training and dietary intervention is more effective than either alone and combined exercise. For example, Jakicic *et al*. compared the effects of different durations (1,000 kcal/week vs. 2,000 kcal/week) and intensities (moderate vs. vigorous) of exercise on 12 month weight loss.^63^ All participants (201 sedentary, obese women) were instructed to reduce caloric intake to between 1,200 and 1,500 kcal/day. After one year of intervention, all four groups had a similar weight loss of approximately 6 kg, with mean losses ranging from 6.3 (SD: 5.6) kg in the moderate-intensity/moderate-duration group to 8.9 kg (SD: 7.3) in the high-intensity/high-duration group.^63^ These finding would suggest that higher levels of PA have no effect on short- or long-term weight loss. However, *post hoc* analysis revealed that those who exercised ≥300 min/week (expending ∼2,000 kcal/week) maintained weight losses nearly three times as great as participants whose activity was <150 min/week (expending <1,000 kcal/week).^64^ The purpose of a recent study by Jakicic *et al*. was to examine the effect of different prescribed doses of moderate–vigorous intensity PA on body weight in overweight adults.^65^ The mean change on body weight resulting from an intervention that promotes 150–300 min/week of moderate-intensity PA with no reduction in energy intake was <2.0 kg. Participants classified as reducing body weight by more than 3% over a period of 18 months increased PA by 162 min/week above baseline levels, whereas those participants categorized as being within 3% of their initial body weight, or gaining more than 3% of their body weight, increased PA above baseline levels by 78.2 and 74.7 min/week, respectively. The magnitude of weight loss of those classified as reducing weight by more than 3% was 5.4 kg (SD: 2.6), corresponding with weight loss of 7.4% (SD: 3.6) of initial body weight. These individuals also changed their eating behaviors, which in combination with PA, resulted in the significant decrease in weight. In this context, Wadden *et al*. reported in his recent review new developments in diet, PA, and behavior therapy. Lifestyle modification, also referred to as behavioral weight control, produces a 7–10% reduction in initial weight for obesity.^66^

## Weight loss maintenance

The importance of PA for improving long-term weight loss and minimizing weight regain is supported by the systematic review conducted for the 2009 American College of Sports Medicine Position Paper.^16^ Based on this review, weight maintenance is improved with PA >250 min/week, which is consistent with the results of empirical studies.^63^,^64^,^67^ Jeffery *et al*. evaluated the efficacy for long-term weight loss of recommendations for much higher PA than those normally used in behavioral treatments.^67^ Overweight men and women were randomly assigned to either standard behavior therapy (expending 1,000 kcal/week), or to a high PA treatment (expending 2,500 kcal/week). There were no differences for weight loss between groups at six months, but there were significant differences at 12 and 18 months of follow-up with the 2,500 kcal/week group showing significantly greater weight losses (6.7 ± 8.1 kg vs. 4.1 ± 7.3 kg). Thus, this study indicates that greater levels of PA provided significantly lower levels of weight regain. The simplest explanation is that increased PA helps to maintain energy balance. However, metabolic adaptation—defined as the relative higher (or lower) metabolic rate as a function of the new body weight and composition achieved after weight loss—is also observed under resting conditions, on which there is no PA-dependent energy expenditure. For example, resting energy expenditure decreased 3–4 kcal/kg of fat-free mass per day in subjects after losing 10–20% of their body weight.^68^ Thus, maintenance of a reduced body weight is associated with compensatory changes in energy expenditure, supporting the hypothesis that skeletal muscle is the major “effector” organ for changes in energy output that favor the regain of loss weight.^69^ Importantly, multiple short bouts of activity (≥10 min) throughout the day are as effective as one long bout (>40 min) for achieving weight loss.^70^

## Implications of RT

Although it is clear that aerobic endurance training (AET) stimulates postexercise energy expenditure and is associated with much greater energy expenditure during the exercise session than RT,^71^ studies have shown that regular RT is effective in promoting weight control in obese persons. Combining RT with AET has been shown to be superior for body weight and fat loss and to result in greater lean body mass when compared to AET alone.^72^,^73^ Although the addition of RT to dietary restriction has been shown to have limited effectiveness in reducing body weight or total body fatness, compared with what can be achieved through dietary intervention alone, the addition of RT to a regimen of caloric restriction results in a preservation of lean body mass compared to dieting alone, which may in turn increase resting metabolic rate (RMR).^74^,^75^ RT stimulates increased muscle protein turnover^76^ and actually has a dual impact on RMR. First, as a chronic response, RT results in greater muscle mass that necessitates more energy at rest for ongoing tissue maintenance. Theoretically, a gain of 1 kg in muscle mass should result in a RMR increase of approximately 21 kcal/kg of new muscle. For example, a difference of 5 kg in lean body mass translates to a difference in energy expenditure of 100 kcal/day (equivalent to 4.7 kg fat mass per year).^77^ Thus, RT does not enhance weight loss but may increase lean body mass and loss of fat mass. Indeed, four months of RT may increase lean body mass by 0.5–3.2 kg,^78^,^79^ reduce fat mass by 1.3–3.8 kg,^78^,^80^ and increase RMR by 7%,^81^,^82^ Second, as an acute response, RT causes microtraumata that require relatively large amounts of energy for muscle remodeling processes that may persist for six days after the training session.^83^ It is incontestable that AET is a powerful inducer of mitochondrial biogenesis in muscle.^84^ However, based on the mitochondrial theory of aging (biological aging is typically associated with a progressive increase in body fat mass, especially visceral fat, and a loss of lean body mass),^85^ RT may serve as a countermeasure of age-associated mitochondrial dysfunction by reducing potentially damaging compounds to mitochondria resulting from reactive oxygen species as natural consequence of age-related sarcopenia.^86^,^87^

## PA, fitness, and MS

The pathogenesis of MS is multifactorial and progressive. The risk factors of MS are of metabolic origin and consist of abdominal adipose tissue accumulation, atherogenic dyslipidemia, elevated plasma glucose, elevated blood pressure (BP), and a prothrombotic and proinflammatory state. The major risk factors are abdominal obesity and insulin resistance accompanied by increased risk for CVD and T2D. Furthermore, aging, physical inactivity, endocrine, and genetic factors exacerbate the MS.^88^

PA is considered to reduce the risk of developing MS and is an important component of CVD prevention. Cross-sectional studies found an inverse gradient between amount of PA and MS.^9^,^45^,^89^–^91^ Guidelines support that at least 150 minutes of moderate-intensity PA per week is associated with a lower prevalence of MS.^16^ The lowest prevalence can be seen in those individuals performing sports activity with high intensity and regularity (more than two hours weekly), whereas everyday activities such as walking and cycling may not have an additional influence.^9^ Further, prospective studies show a strong inverse dose response between cardiorespiratory fitness and risk of developing MS.^92^–^94^ The EPIC-Norfolk prospective population study investigated the association among PA, MS, and the risk of future CHD and mortality due to CHD in 10.134 middle-aged men and women.^95^ The prevalence of MS was 37.6% in men and 30.2% in women. CHD risk associated with MS was substantially lower among participants who were physically active. There was no longer a significant difference in CHD event rate between men with MS who were active and men without MS who were inactive. The authors found evidence for significant effect modification such that PA affected the association between MS and CHD risk.^95^ To date, there are few studies that have examined the role of RT in the prevention of MS.^96^–^98^ In both cross-sectional and longitudinal reports from the Aerobic Center Longitudinal Database, higher levels of muscular strength were associated with lower risk of MS.^97^,^98^

Although only few studies have examined the efficacy of different modes of exercise in the reversal of the clinical diagnosis of MS,^99^ numerous studies and systematic reviews have reported the benefits of AET or RT on components of MS, such as abdominal obesity, BP, blood lipids, and insulin resistance.^100^–^102^ The following part focuses on supervised exercise training studies for the purpose of determining the role of PA on changes in MS risk factors among obese individuals.

## PA and abdominal fat

Adipose tissue is a major endocrine organ, secreting substances such as adiponectin, leptin, resistin, tumor necrosis factor α, interleukin 6, and plasminogen activator inhibitor-1 that may play a critical role in the pathogenesis of the MS.^103^ Visceral (intra-abdominal) adipose tissue (VAT) compared to total body fat correlates significantly better with triglycerides, systolic and diastolic BP, and is expected to decrease the sensitivity of target tissues to insulin.^104^,^105^

There are a number of well-designed studies that have studied the effects of exercise on VAT. A recent meta-analysis summarized the effects of AET and progressive RT for beneficial VAT modulation.^106^ These data suggest that AET—even below current recommendations for obesity management—is effective in lowering VAT, while RT itself failed to induce significant reduction in VAT when compared with control. There are several possible reasons for this discrepancy. It has been suggested that AET has specific effects on decreasing VAT as it may lead to increased sympathetic tonus, thereby increasing lipolysis especially in abdominal fat.^106^,^107^ Especially high-intensity AET can lead to chronic increases in 24-hour growth hormone release, which acts to stimulate adipose tissue directly via hormone sensitive lipase and also indirectly by enhancing insulin sensitivity.^108^ Further, AET involves continuous activity of multiple large muscle groups, whereas RT involves isolated, brief activity of single muscle groups. However, combining RT with AET has been shown to be superior for VAT loss and to result in greater lean body mass when compared to AET alone.^72^,^73^

A recent systematic review focused on the potential and unique effect of RT on VAT and specific biomarkers of inflammation.^109^ Although the results show only a slight decrease in VAT with RT as the sole intervention, the clinical significance can be gauged by studying large prospective intervention studies examining the correlations between changes in VAT with exercise training and variables of metabolic risk. In STRRIDE, data suggests that the reduction of as little as 11 cm^2^ in VAT is significantly related to changes in low-density lipoprotein (LDL) particle number, LDL size, and insulin sensitivity.^110^ Data from STRRIDE also revealed that a higher amount of AET resulted in greater reductions in measures of central obesity but there was no dose–response relationship between intensity of exercise and changes in VAT.^58^ Similarly, a 2007 review by Ohkawara *et al*. found that there is a dose–response relationship between amount of exercise and changes in VAT in obese subjects without metabolic-related disorders.^111^ A significant VAT reduction was observed from about 10 metabolic equivalents (METs) x hours/week, and if obese subjects without metabolic dysfunction practiced AET, the degree of VAT loss could be directly attributed to the aerobic exercise-amount (i.e., to reduce 10% of VAT in 10 weeks, 27 METs x hours/week is required).^111^ In contrast, on the basis of recent data by Ismail *et al*., although there was a significant relationship between mean weight loss and VAT reduction (*r*^2^ = 0.17, *P* < 0.05), they found no evidence to suggest a relationship between total weekly exercise volume or mean intensity and VAT reduction.^106^ In spite of the trend that the more weight is lost, the more VAT is reduced,^111^ a significant reduction of VAT, which occupies less than 5% of body weight, may also occur without significant weight loss.^58^,^59^ These results provide evidence of the usefulness of AET for VAT reduction. Unfortunately, it is also apparent that in sedentary middle-aged men and women, short periods of physical inactivity lead to significant weight gain, substantial increases in VAT, and further metabolic deterioration.^112^ One study reported a 38% increase in VAT after a one-year follow-up of a diet-induced weight loss program in a group not compliant to regular exercise during the follow-up period, but there were no significant changes in VAT in either an AET or RT group that adhered to regular exercise training this same time period.^113^ Thus, both training modalities prevented the regain of VAT. In a similar design, VAT increased 21.3 ± 5.3% over a two-year period in overweight and obese premenopausal women in a control group, but only 7.0 ± 5.1% during the same time period in a RT group.^114^ It was concluded that RT attenuates VAT increases occurring over time in women. It seems that RT has the potential to reduce VAT through both immediate effects (during weight loss or weight maintenance) and delayed effects (during weight regain).

## PA and blood lipids

The association between serum cholesterol and CVD outcomes is well documented. In particular, LDL and apolipoprotein B have been correlated with the development of CHD- and CVD-related events.^115^ Published data show a correlation between PA and triglyceride reduction,^116^ apolipoprotein B reduction,^117^ high-density lipoprotein (HDL) increase,^118^ and change in LDL particle size.^116^,^119^ In fact, studies indicate that regular exercise training does not significantly reduce total cholesterol or LDL independent of weight loss.^116^ However, data do suggest that regular PA may change LDL particle size, even when total LDL concentration remain constant.^116^,^119^ Kraus *et al*. found that 25 min of daily AET increased mean LDL particle size irrespective of training intensity or weight loss.^116^ Thus, exercise training appears to reduce CVD risk, in part, because of increases in LDL particle size rather than significantly lowering LDL concentration.

Although there are conflicting data regarding the effect of regular exercise on atherogenic lipoproteins, there is strong evidence for changes in apolipoprotein B, HDL, and triglycerides with regular exercise. Longitudinal studies have shown regular exercise to reduce apolipoprotein B up to 20%.^117^,^120^ As for HDL and triglycerides, one meta-analysis by Carroll and Dudfield showed that long-term, moderate-intensity exercise training increases HDL and lowers triglycerides even in the absence of weight loss.^100^ With 30–60 min of moderate-intensity AET three to five times per week, HDL levels were noted to increase by 0.05 mmol/L (95% CI: 0.03–0.06 mmol/L), and triglyceride levels decreased by 0.21 mmol/L (95% CI: –0.29–0.14 mmol/L). Others have shown that HDL increases by 0.008 mmol/L per mile of running per week.^118^ On the other hand, a meta-analysis of 13 trials of at least eight weeks duration among overweight and obese adults indicated that AET reduced triglycerides by 11% (–19.3 ± 5.4 mg/dL; 95% CI: –30.1 to –8.5 mg/dL), with minimal effects of total cholesterol, HDL and LDL cholesterol.^121^ In a meta-analysis of 61 study groups, Leon and Sanchez reported that changes in total cholesterol, LDL cholesterol, and triglycerides were moderately correlated with loss of body mass, but changes in HDL cholesterol were not.^122^ These results are similar to those reported in the HERITAGE Family Study, where changes in blood lipids were correlated with changes in fat mass, but not with changes in aerobic fitness after 20 weeks of supervised AET.^123^

The evidence from a very recent review indicates very little effect of PA on the blood lipid profile in the absence of dietary restriction.^102^ However, there is mounting evidence for changes in HDL and triglycerides, as well as of alteration in LDL particle size with exercise. Thus, exercise training has the potential to decrease atherogenicity, but the ideal exercise mode, intensity, and dose response that yields the maximal beneficial adaptations is in dispute. The purpose of a pilot study was to compare continuous moderate exercise training (70% of maximum heart rate) and high-intensity aerobic interval training (90% of maximum heart rate) on variables associated with cardiovascular function and prognosis in patients with the MS.^99^ Aerobic interval training increased HDL cholesterol by 25% but remained unaltered in the moderate exercise and control group. Neither training program changed the levels of LDL, total cholesterol, or triglycerides. Recently, non-HDL cholesterol concentration has become a powerful predictor of CVD morbidity and mortality.^124^,^125^ This is due to the fact that non-HDL cholesterol contains all known atherogenic lipid particles (lipoprotein (a), LDL, intermediate density lipoprotein, and very LDL).

**Figure 4:**
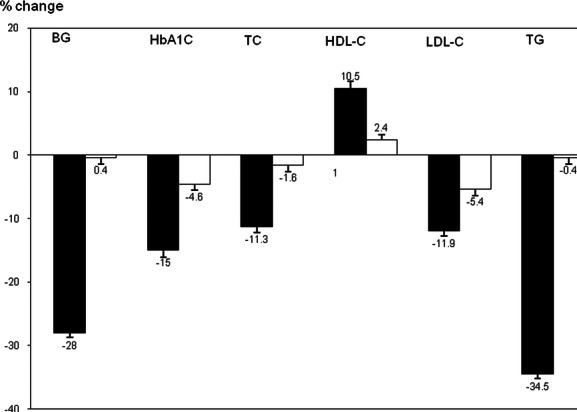
Percentage change in metabolic parameters after four months RT (black) or AET (white) in patients with T2D. Whiskers represent standard deviation. Source from work by Cauza *et al*.^78^

At present, a small amount of conflicting data exists on the effects of RT on blood lipid levels. Kelley and Kelley conducted a meta-analysis and concluded that RT reduced total cholesterol by 2.7% (95% CI: –4.6% to –0.8%), non-HDL cholesterol by 11.6% (95% CI: –20.9% to –4.7%), LDL cholesterol by 4.6% (95% CI: –8.4% to –0.8%), triglyceride by 6.4% (95% CI: –11.4% to –1.4%), while increasing total HDL cholesterol by 1.4% (95% CI: –2.4 to 5.2), with no significant overall changes for HDL cholesterol.^126^ The specific aims of a recent study were to quantify the effects of 12 weeks of RT as well as a single session of RT on lipids and lipoproteins in 21 obese, postmenopausal women.^127^ The key findings of this study were that a single bout of RT did not modify circulating lipid and lipoprotein–cholesterol concentration 24 hours postexercise. Although 12 weeks of RT provided no significant changes in body mass or body composition, total cholesterol, LDL cholesterol, non-HDL cholesterol and HDL_3_ cholesterol concentration were 23.6%, 28.5%, 27.0%, and 24.1%, respectively lower in the RT group when compared to the control group following 12 weeks of RT.^127^ It was the aim of one of our in-house studies to compare the effects of a four-month RT versus AET program on metabolic control in subjects with T2D.^78^ We found improvements in the atherogenic lipid profile after four months of progressive RT, whereas the effects of AET on metabolic parameters were only modest (Fig. 4). In the RT group, we observed a significant reduction of triglyceride levels (from 229 ± 25 mg/dL to 150 ± 15 mg/dL, *P* = 0.001), total cholesterol (from 207 ± 8 mg/dL to 184 ± 7 mg/dL, *P* < 0.001), and LDL cholesterol (from 120 ± 8 mg/dL to 106 ± 8 mg/dL, *P* = 0.001), and a significant increase in HDL cholesterol (from 43 ± 3 mg/dL to 48 ± 2 mg/dL, *P* = 0.004). Importantly, these observations were made in absence of dietary changes during the training period. The positive alteration in the lipid profiles must be largely due to the changes in body composition as a result of RT. There was a strong correlation between changes in lean body mass (from 49.4 ± 1.8 kg to 52.6 ± 1.7 kg) and changes in total cholesterol (*r* = 0.44, *P* < 0.05) and triglyceride levels (*r* = 0.46, *P* < 0.05). The large percentage improvements in the atherogenic lipid profile in the latter study may have a clinical significance. In clinical trials examining effects of statins on the risk of CHD morbidity and mortality, an approximate 30–40% reduction in risk is observed with the majority of the effect directly related to the absolute reduction in LDL cholesterol.^128^ Moreover, a meta-analysis of four large prospective studies consistently showed that for every 1 mg/dL (0.026 mmol/L) decrease in plasma levels of HDL cholesterol there was a 2–3% increase in the risk of CHD, independent of other risk factors, including plasma LDL cholesterol.^129^ Hurley *et al*. concluded that this finding of reductions in blood triglyceride levels reported in the above exercise studies may have important clinical implications because the criteria used to diagnose the MS by both the International Diabetes Federation and the NIH's ATPIII use elevated triglyceride, but not elevated total cholesterol or LDL cholesterol levels.^130^

## PA and glucose metabolism

Disturbances in glucose and insulin metabolism may not be a normal characteristic of aging, but rather associated with obesity and physical inactivity.^131^ Recent evidence from the Emerging Risk Factors Collaboration demonstrates that glucose levels exceeding 100 mg/dL are a key risk factor for life expectancy.^132^ Lifestyle change, including PA and weight loss, is the central strategy in T2D prevention. The Finnish Diabetes Prevention Study demonstrated 58% lower incidence of T2D over four years in participants who exercised an average of 210 min/week of moderate to strenuous intensity compared to controls.^8^ A similar risk reduction of 58% was reported in the U.S. Diabetes Prevention Program that prescribed 150 min/week of moderate activity exercise and dietary intervention designed to induce a 7% weight loss.^133^ The Look AHEAD study was initiated to examine the role of diet and intensive lifestyle intervention (ILI) on risk of CVD in individuals of T2D.^134^ At one year, participants in the ILI lost 8.6% (SD: 6.9%) of initial weight in comparison with 0.7% (SD: 4.8%) in the support and education group. The ILI produced significantly greater improvements in hemoglobinA_1c_ (HbA_1c_) (26.4 ± 1.0% vs. 5.4 ± 1.1%), fitness, and numerous measures of CVD risk compared to control.^135^

PA is associated with improvements in glucose and insulin metabolism.^136^,^137^ Glycemic control has traditionally been the primary focus of exercise training studies in patients at risk or with T2D. Daily PA has been shown to be a mediator of glycemic control even without diabetes.^138^ Moreover, a single bout of exercise can substantially reduce the prevalence of hyperglycemia for the following 24 hours.^139^ Beneficial effects have been shown with both AET,^140^ RT,^78^,^79^ or a combination of both modes of training.^80^ These improvements in glycemic control may result in reductions in T2D medications.^78^ The mechanisms responsible for these exercise-induced benefits are complex and include improvements in insulin sensitivity,^141^ increases in muscle GLUT4 number and function,^142^ increases in muscle capillarization, and blood flow.^143^ These adaptations are strongly influenced by energy expenditure.^144^

Blood glucose decreases during any PA are related to the intensity and duration of the exercise, preexercise control, and state of exercise training.^80^,^136^,^137^,^140^ Although PA of any intensity generally enhances uptake of circulating glucose, and stimulates fat oxidation, more prolonged or intense activity usually enhances acute insulin action for longer.^140^,^145^ Recently, low-volume, high-intensity AET (10 × 1 min at 90% HR_peak_) was shown to rapidly improve glucose control and metabolic health in adults with T2D.^146^ Similarly, another study has shown that uphill walking (4 × 4 minutes at 90–95% HR_peak_ with three minutes active recovery in between bouts) was more effective in reversing the risk factors of the MS than moderate intensity exercise.^99^ Indeed, a meta-analysis found that exercise intensity is more important in improving insulin sensitivity than duration.^147^ However, for a large majority of people at risk or with T2D, moderate intensity of exercise may be more appropriate, better tolerated, and result in greater exercise adherence.^148^ Moreover, even light-intensity PA is associated with blood glucose reductions, whereas sedentary time is unfavorable associated with increased levels.^36^

Similar to the intensity of exercise, the optimal duration of exercise in patients with T2D or prediabetes remains undefined. A meta-analysis by Umpierre *et al*. has recently shown that engaging in structured exercise training of more than 150 min/week results in greater glycemic benefits, thus total exercise dose may be important.^149^ Based on the actual position statement from Exercise and Sport Science Australia, it is recommended that patients with T2D or prediabetes accumulate a minimum of 210 min/week of moderate-intensity exercise or 125 min/week of vigorous intensity exercise with no more than two consecutive days without training.^148^ Most exercise interventions in adults with T2D have used a frequency of three times per week.^78^,^136^,^140^ Exercise dose (as the product of exercise intensity, duration and frequency) has been shown to be significantly related to improvements in insulin action, while age, gender and frequency may not.^150^ The purpose of a recent crossover study was to investigate the impact of daily exercise versus exercise performed every other day on glycemic control in T2D patients.^151^ Subjects were studied for three days under strict dietary standardization. Blood glucose homeostasis was assessed by continuous glucose monitoring over 48 hours during which subjects performed no exercise (control) or 60 min of cycling exercise (50% maximal workload capacity) distributed either as a single session performed every other day or as 30 min of exercise performed daily. A short 30-min session of moderate-intensity AET significantly reduces the prevalence of hyperglycemia throughout the subsequent day in T2D patients. It appears that as long as total work is being matched, daily exercise does not further improve glycemic control compared with exercise performed every other day.^151^

Recent reviews indicate that RT is considered a potential adjunct in the treatment of metabolic disorders by decreasing known major risk factors for MS.^101^,^130^,^152^,^153^ An overview of how RT versus AET may influence age-related physiological changes influencing metabolic risk is presented in Table 1. RT can improve glycemic control and insulin sensitivity, likely even more so than AET.^78^,^79^ It is possible that an increase in lean body mass after RT may be an important mediator of the improved glycemic control. An increase in the number of GLUT4 transporters is discussed specifically, because the transporter protein GLUT4 expression at the plasma membrane is related to fiber volume in human skeletal muscle fibers.^154^ However, increased muscle mass was not associated with improvement in glycemic control in one of our in-house study.^155^ One possible reason is that improvement in glycemic control is not only dependent on muscle mass change but also the consequence of intrinsic alterations in the muscle.^142^ Furthermore, reductions in visceral fat and inflammation with RT may improve glucose uptake and reduce diabetes risk.^73^,^74^

**Table 1:** Comparison of the effects of aerobic endurance training to resistance training on metabolic health variables

Variable	Effects of RT	References	Effects of AET	References
Aging muscle				
Muscle strength	↑↑↑	78–80	↔	78–80
Mitochondrial biogenesis	↑	86, 87	↑↑	84, 146
GLUT4 content	↑↑	142, 154	↑	146
Body composition				
Lean body mass	↑↑	78–80	↔	78, 80
Percent body fat	↓↓	78–80	↓↓	58, 59
Visceral abdominal fat	↓↔	106, 109	↓	59, 106, 110
Glucose metabolism				
Glycosylated hemoglobin	↓↓	78, 79, 101, 140, 143	↓↓	79, 136, 140
Insulin sensitivity	↑↑	78, 79	↑↑	79, 141
Lipids and lipoproteins				
HDL cholesterol	↑↔	78, 101, 126	↑	100, 116, 118
LDL cholesterol	↓↔	78, 101, 126	↔	100, 117
Triglycerides	↓↔	78, 101, 126	↓↓	100, 116, 121
Apolipoprotein B	↓	171	↓	117, 120
Inflammation				
Adiponectin	↑↔	109	↑	172
Leptin	↓↔	109	↓↔	174
Interleukin-6	↓↔	109	↓↔	174
Tumor necrosis factor-α	↔	109	↓↔	174
C-reactive protein	↓↓	109	↓↔	173, 174
Basal metabolic rate	↑↑	81, 82	↑	71

AET, aerobic endurance training; RT, resistance training. ↑ indicates values increase; ↓ indicates values decrease; ↔ indicates values remain unchanged; one arrow, small effect; two arrows, moderate effect; three arrows, large effect.

In a recent meta-analysis, aerobic, resistance, and combined exercise training were found to be associated with HbA_1c_ reductions of 0.67% (95% CI: –0.84% to –0.50%) following 12 or more weeks of training.^149^ In another meta-analysis including 10 supervised resistance exercise studies, RT reduced HbA_1c_ by 0.48% (95% CI: –0.76% to –0.21%).^101^ This is not unimportant since a one unit increase in HbA_1c_ is associated with a 28% increase in mortality^156^ and a one unit decrease with a 37% reduction in microvascular complications.^157^ Improvements in glycemic control can result from a variety of different training intensities.^101^ However, because of reduced adherence and training intensity, home-based RT is less effective for maintaining glycemic control than supervised RT.^158^ Theoretically, both AET and RT should be combined in the exercise prescription for T2D and prediabetes. Recent research has identified that combining both forms of exercise of an equal caloric expenditure (12 kcal/kg/week) among combined and separate AET and RT groups may lead to greater glycemic control benefits that was not found in either type of training alone.^80^ It is recommended that two or more RT sessions per week (2–4 sets of 8–10 repetitions) should be included in the total 210 or 125 min of moderate or vigorous exercise, respectively.^148^

## PA and BP

Obese individuals have an elevated risk of having high BP.^2^,^159^ It is widely accepted that PA on a regular basis has an antihypertensive effect.^160^,^161^ Indeed, regular exercise training is able to reduce heart rate, improving the sensitivity of aortic baroreceptors, which contributes to a better regulation of BP.^162^ Other mechanisms include the decrease in peripheral arterial resistance caused by vasodilatation.^162^ Some trials have reported that change in BP during exercise is strongly associated with reduction in insulin resistance and insulin levels in hypertensive patients.^163^ A meta-analysis of 72 trials revealed that BP responses to AET were more pronounced in hypertensive study groups (–6.9 mmHg; 95% CI: –9.1, –4.6 mmHg/–4.9 mmHg; 95% CI: –6.5, –3.3 mmHg) compared to normotensive groups (–2.0 mmHg; 95% CI: –3.0, –0.9 mmHg/–1.6 mmHg; 95% CI: –2.3, –1.0 mmHg), respectively.^161^ An earlier review that included 54 randomized controlled trials reported that AET was associated with a significant reduction in mean systolic and diastolic BP (–3.84 mmHg; 95% CI: –4.97, –2.72 mmHg/–2.58 mmHg; 95% CI: –3.35, –1.81 mmHg, respectively).^160^ A reduction in BP was associated with AET in hypertensive and normotensive participants and in overweight and normal-weight participants.^160^ Similarly, Hu *et al*. has shown that BP responses to exercise are not related to baseline BMI.^159^ A meta-analysis in T2D patients showed that AET reduce systolic BP of about –4.16 mmHg.^164^ Such reduction for mean systolic BP is not insubstantial since a reduction of as little as 3 mmHg in systolic BP has been estimated to reduce CHD by 5–9%, stroke by 8–14%, and all-cause mortality by 4%.^165^

Considering the benefits of AET on BP, we pose an important question: How much exercise is needed to confer such benefits? In the past, Hagberg *et al*. indicated that AET significantly reduced BP in approximately 75–80% of groups with hypertension, with weighted mean reduction of 10.6/8.2 mmHg, and reported lower intensities (<70% VO_2max_) to be more effective than higher intensities (>70% VO_2max_) in lowering high BP.^166^ On the other hand, meta-analytic reviews by Whelton *et al.* and Cornelissen and Fagard, both reported no influence of exercise intensity on BP reduction following exercise treatment.^160^,^167^ Even though BP reductions were found for one and two days/week, a frequency of three days/week has been considered to be the minimal frequency for BP reduction.^168^ The range of exercise duration reported in the literature has been 10–60 minutes.

The American College of Sports Medicine position on exercise and hypertension concluded that RT elicits significant reductions in BP in normotensive, prehypertensive, and hypertensive patients.^168^ Data were pooled from nine randomized controlled trials in a meta-analysis on the effects of RT on BP.^169^ The authors concluded that RT reduced systolic BP by 3.2 mmHg (95% CI: –7.1–0.7 mmHg) and diastolic BP by 3.5 mmHg (95% CI: –6.1 to –0.9 mmHg).^169^ The purpose of a recent meta-analysis by Cornelissen *et al.* of 33 study groups was to investigate the effects of RT on BP and other cardiovascular risk factors in healthy adults.^170^ Overall, RT induced a significant BP reduction in 28 normotensive or prehypertensive study groups (–3.9 mmHg; 95% CI: –6.4, –1.2 mmHg/–3.9 mmHg; 95% CI: –5.6, –2.2 mmHg), whereas the reduction was not significant for the five hypertensive study groups (–4.1 mmHg; 95% CI: –0.6, 1.4 mmHg/–1.5 mmHg; 95% CI: –3.4, 0.4 mmHg). When study groups were divided according to the mode of training, isometric handgrip training in three groups resulted in a larger decrease in BP (–13.5 mmHg; 95% CI: –16.5, –10.5 mmHg/–6.1 mmHg; 95% CI: –8.3, –3.9 mmHg) than dynamic RT in 30 groups (–2.8 mmHg; 95% CI: –4.3, –1.3 mmHg/–2.7 mmHg; 95% CI: –3.8, –1.7 mmHg).^170^ Based on our recent meta-analysis in patients with T2D, we show that the effect of RT on resting BP seems to be dose dependent, since decreases in resting systolic BP (–6.2 mmHg; 95% CI: –11.4, –1.0 mmHg) were more pronounced when the RT program was of high volume.^101^ Furthermore, relatively modest increases in RT frequency had hypotensive effects, since resting BP were further reduced when exercising three times per week compared to twice a week.^101^

## Conclusions

The information presented in this review provides strong support for the recommendation that PA should be an integral component in the prevention and treatment of obesity and MS risk factors. Current guidelines suggest that adults should accumulate about 60 min of moderate-intensity PA daily to prevent unhealthy weight gain. Hu noted two reasons why greater PA is required to maintain body weight. First, the current food environment encourages excess caloric intake and positive energy balance. Second, the lifestyle during nonleisure-time PA has become increasingly sedentary, a trend that will continue.^54^ Studies have shown that lifestyle activity, which involves increasing energy expenditure throughout the day, without concern for the intensity or duration of the activity, is as effective for weight control as more traditional programmed activity. Overweight and obese individuals should be encouraged to walk an extra 2,000–6,000 steps per day, which would expend about 100–300 kcal more. Although it is clear that AET is associated with much greater energy expenditure during the exercise bout than RT, some studies have shown that combining RT with AET during dietary energy restriction is remarkably effective in promoting weight loss and preventing weight gain compared to AET alone. The reason is that an obese person who restricts energy to lose weight and also performs AET would not have an anabolic stimulus to preserve lean body mass from the aerobic exercise. RT aids in retaining skeletal muscle mass that is metabolically active. Furthermore, more intense PA, specifically RT, has been shown to increase in the active muscle GLUT4 amount, alleviate insulin resistance and to promote a more favorable lipid profile in patients with T2D.^171^ Based on a recent meta-analysis, we show that RT has the power to significantly reduce resting levels of serum C-reactive protein (CRP) by 25% (–0.23 mg/L; 95% CI: –0.38, –0.07 mg/L) independently from weight loss in sedentary healthy or overweight/obese adults and tends to improve adiponectin and leptin profile with intensities equal or greater than 80% of one repetition maximum.^109^,^172^ It seems that PA alone, such as brisk walking, does not result in significant reductions in CRP,^173^ and it is possible that changes in body mass (fat loss, lean body mass increase) with long-term high-intensity (preferably mixed) training, in addition to daytime PA, is required to obtain a significant anti-inflammatory effect.^174^,^175^ As such, combining RT with AET is highly recommended in the management of obesity and metabolic disorders when compared to RT or AET alone.
